# Developmental nicotine exposure affects larval brain size and the adult dopaminergic system of *Drosophila melanogaster*

**DOI:** 10.1186/s12861-018-0172-6

**Published:** 2018-06-14

**Authors:** Melanie Morris, Ariel Shaw, Madison Lambert, Haley Halperin Perry, Eve Lowenstein, David Valenzuela, Norma Andrea Velazquez-Ulloa

**Affiliations:** 1 School of Medicine, University of Washington, Seattle, USA; 2 Biochemistry, Cell and Molecular Biology Program, Lewis & Clark College, Portland, USA; 3 Biology Department, Lewis & Clark College, Portland, USA; 4 Madison High School, Portland Public Schools, Portland, USA

**Keywords:** Development, Nicotine, *Drosophila*, Dopamine, Brain, *Dα7*

## Abstract

**Background:**

Pregnant women may be exposed to nicotine if they smoke or use tobacco products, nicotine replacement therapy, or via e-cigarettes. Prenatal nicotine exposure has been shown to have deleterious effects on the nervous system in mammals including changes in brain size and in the dopaminergic system. The genetic and molecular mechanisms for these changes are not well understood. A *Drosophila melanogaster* model for these effects of nicotine exposure could contribute to faster identification of genes and molecular pathways underlying these effects. The purpose of this study was to determine if developmental nicotine exposure affects the nervous system of *Drosophila melanogaster*, focusing on changes to brain size and the dopaminergic system at two developmental stages.

**Results:**

We reared flies on control or nicotine food from egg to 3rd instar larvae or from egg to adult and determined effectiveness of the nicotine treatment. We used immunohistochemistry to visualize the whole brain and dopaminergic neurons, using tyrosine hydroxylase as the marker. We measured brain area, tyrosine hydroxylase fluorescence, and counted the number of dopaminergic neurons in brain clusters.

We detected an increase in larval brain hemisphere area, a decrease in tyrosine hydroxylase fluorescence in adult central brains, and a decrease in the number of neurons in the PPM3 adult dopaminergic cluster. We tested involvement of *Dα7*, one of the nicotinic acetylcholine receptor subunits, and found it was involved in eclosion, as previously described, but not involved in brain size.

**Conclusions:**

We conclude that developmental nicotine exposure in *Drosophila melanogaster* affects brain size and the dopaminergic system. Prenatal nicotine exposure in mammals has also been shown to have effects on brain size and in the dopaminergic system. This study further establishes *Drosophila melanogaster* as model organism to study the effects of developmental nicotine exposure. The genetic and molecular tools available for *Drosophila* research will allow elucidation of the mechanisms underlying the effects of nicotine exposure during development.

## Background

Tobacco use has been linked to multiple illnesses and contributes to about seven million deaths each year [1, 2]. According to the 2013 National Survey of Drug Use and Health, 15.4% of pregnant women reported recent cigarette use in the United States of America and this percent has not significantly decreased over the last decade [3]. This is in light of the fact that smoking during pregnancy has been shown to be harmful and have multiple consequences on the offspring, including a decrease in developmental viability and perturbations in the neural architecture of the brain, coupled to abnormal behavioral outcomes later in life [1, 4–12].

Although tobacco has thousands of compounds, nicotine has been identified as the addictive substance [13]. Pregnant women may also be exposed to nicotine in other ways such as e-cigarettes, which are perceived as safer than tobacco cigarettes, or through nicotine replacement therapy, which is a treatment to quit smoking approved by the Federal Drug Administration and commonly offered as a cessation intervention [14–16]. There is little evidence that e-cigarettes are effective for smoking cessation and it has been shown that e-cigarette exposure during development leads to behavioral changes in rodents [17, 18].

The effects of developmental nicotine exposure in rodents recapitulate known effects of tobacco exposure in humans and include lower birth weight, delayed development, and alterations of the cholinergic system [4, 5, 7, 19–22]. These effects have also been documented in *Drosophila melanogaster* [23]. Prenatal nicotine exposure also leads to structural changes in the nervous system, including differences in brain size, and other alterations to dendrite, spines and specific regions of the CNS [24–26]. The dopaminergic system, a neurotransmitter system implicated in reward and addiction, is also affected by prenatal nicotine exposure. Dopamine plays a role in normal development of the nervous system, including development of reward pathways [27]. Prenatal nicotine exposure in mammals has been shown to stimulate dopamine release in the fetal forebrain and to affect dopamine levels and turnover, with either decreases or increases depending on the specific region of the dopaminergic system under study [28–34]. Additional knowledge regarding the mechanisms underlying the effects of developmental exposure to nicotine is needed to uncover potential targets for novel medical interventions that could prevent or ameliorate these effects.

Mechanistic research in *Drosophila melanogaster* can be done at a faster rate than could be accomplished in mammalian model systems and can contribute to the elucidation of the mechanisms underlying the effects of developmental nicotine exposure. There is high conservation in basic biological, physiological and neurological properties between *Drosophila melanogaster* and mammals. It has been estimated that between 65 and 75% of human disease-causing genes have a functional homologue in *Drosophila* [35–37].

*Drosophila* has been successfully used to study drug-induced behaviors and the mechanisms of action underlying the responses to acute exposure to ethanol and cocaine in adult flies [38–40]. Research has also been carried out to identify genes involved in the acute and chronic response to nicotine in adult flies [41–47]. A *Drosophila* model could be used to elucidate the mechanisms underlying developmental nicotine’s effects by profiting from the wide array of molecular and genetic tools available in *Drosophila* research [48].

We have recently developed a *Drosophila melanogaster* model that recapitulates several effects of developmental nicotine exposure and shows negative effects of developmental nicotine exposure on normal development [23]. Flies reared on nicotine food had decreased survival, developmental delay, and reduced adult weight with increasing nicotine concentrations. In addition, developmental nicotine exposure decreased adult sensitivity to acute exposure to nicotine and ethanol.

Here we use this *Drosophila* model for developmental nicotine exposure to demonstrate additional effects of nicotine on the nervous system at two developmental stages: 3rd instar larva and adult. The aim of this study was to determine whether developmental nicotine exposure affects brain size or alters the dopaminergic system in *Drosophila melanogaster*. Our data show that developmental nicotine exposure has effects on brain size, dopamine levels and the number of neurons in individual dopaminergic clusters that differ at the larval and adult developmental stages. These data complement the previous characterization of developmental nicotine exposure in *Drosophila melanogaster*.

## Methods

### Drosophila strains and culture

The *w*^*1118*^ Berlin (*w*B) strain used for experiments in this manuscript was gifted by Dr. Ulrike Heberlein. The *Drosophila* nicotinic acetylcholine receptor alpha 7 subunit null strain (*Dα7*^*mut*^), EY6, created by imprecise excision of a P-element insertion, and the precise P-element excision genetic control strain (*Dα7*^WT^), EY5, were a gift by Dr. Amir Fayyazuddin [49]. All fly strains were raised at 25 °C in water baths to keep humidity and vapor pressure more stable. These conditions have yielded homogenous results [23, 50]. Flies were reared on lab-made food with molasses, cornmeal, and yeast medium in a light-dark controlled incubator on a 12 h:12 h light:dark cycle set at 65% humidity.

### Developmental exposure to nicotine and nicotinic acetylcholine receptor blockers

**Fig. 1:**
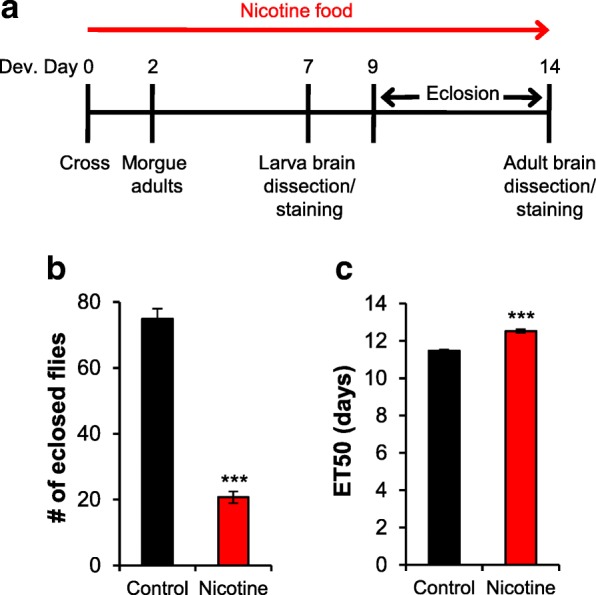
Developmental nicotine treatment affects survival and development time. **a** Schematic showing the experimental design for the nicotine treatment and for when dissections were carried out. Flies were reared on control food (black bars) or food laced with 0.3 mg/ml nicotine (red bars) and the number of flies eclosed was counted from days 9 to 14 after egg laying to estimate survival and the time required for 50% of pupae to eclose (ET50). **b** The number of eclosed flies by day 14 was significantly reduced by the nicotine treatment. **c** The number of days needed for 50% of the flies to eclose was significantly increased by the nicotine treatment. **b**, **c** Samples size was *n* = 64 vials counted for control and *n* = 95 vials for nicotine from 17 independent experiments. Mann-Whitney U-test (**a**) and Student’s t-test (**b**) were used to compare the control versus the nicotine condition

**Fig. 2:**
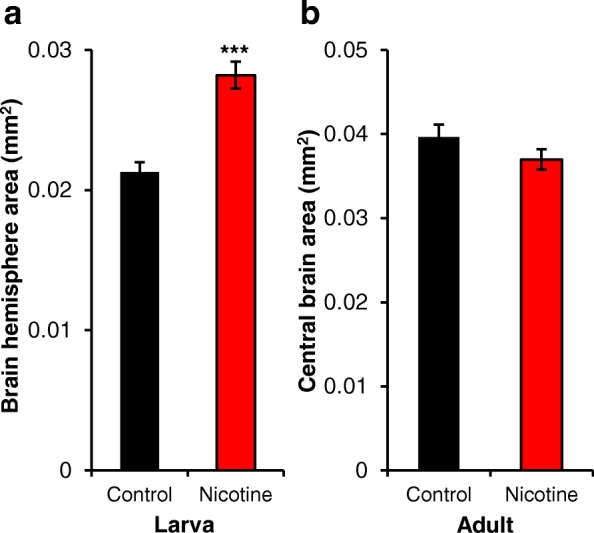
Developmental nicotine treatment increases larval brain hemisphere area. Flies were raised on control food (black bars) or food laced with 0.3 mg/ml nicotine (red bars). Larvae were dissected at the 3rd instar stage of development; adults were dissected 4 days after eclosion. The brains were stained with anti-bruchpilot for visualization. **a** Larval brain hemisphere area was significantly larger in brains from nicotine-exposed larvae. **b** Adult brains had no difference in central brain area between conditions. Sample size for the larval stage was *n* = 35 brain hemispheres for control and *n* = 28 brain hemispheres for nicotine from 6 independent experiments and for adult was *n* = 24 brains for control and *n* = 21 brains for nicotine from 9 independent experiments. Student’s t-test was used to compare the control versus the nicotine condition

**Fig. 3:**
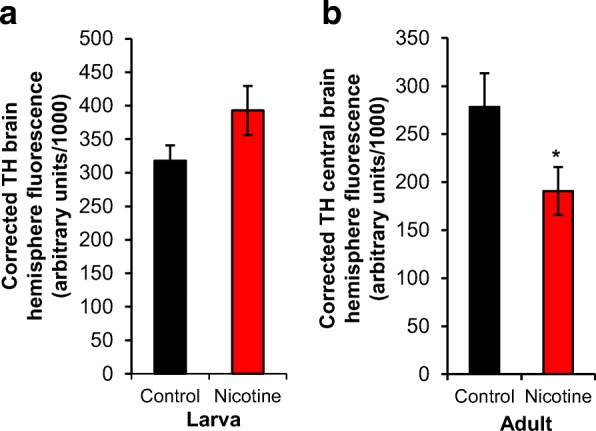
Developmental nicotine exposure decreases TH fluorescence in adult brains. Flies were raised on control food or food laced with 0.3 mg/ml nicotine. Larvae were dissected at the 3rd instar stage of development; adults were dissected 4 days after eclosion. The brains were stained with an antibody against tyrosine hydroxylase (TH). **a** Corrected TH brain hemisphere fluorescence, which normalized the staining fluorescence to background levels, showed that developmental nicotine exposure had no statistically significant effect on TH staining in larval brains. **b** Corrected TH central brain fluorescence was significantly decreased in adult brains of flies exposed to nicotine during development. Sample size was *n* = 22 brain hemispheres for control and *n* = 18 brain hemispheres for nicotine from 5 independent experiments for the larval stage, and *n* = 10 brains for control and *n* = 17 for nicotine from 5 independent experiments for adult. Mann-Whitney U-test (**a**) and Student’s t-test (**b**) were used to compare the control versus the nicotine condition

**Fig. 4:**
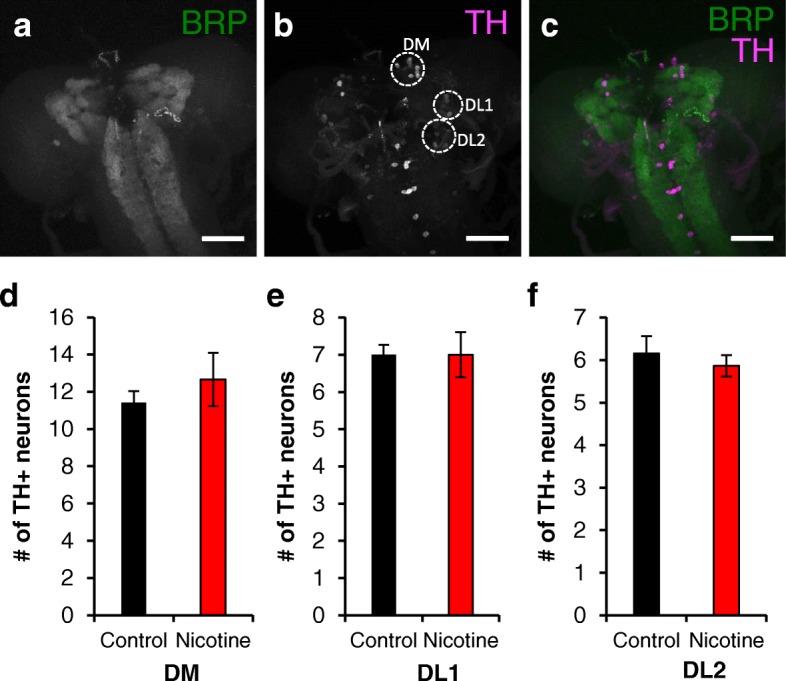
Developmental nicotine exposure does not alter the number of TH+ neurons in larval brains. 3rd instar larvae brains were dissected, immunostained, mounted and imaged on a confocal microscope. **a**-**c** are maximum projection images of a brain from a larva reared in control food and show representative images of larval brains used for analysis. **a** An anti-bruchpilot (BRP) antibody was used as background staining for the whole brain. **b** An anti-tyrosine hydroxylase (TH) antibody was used as marker for dopaminergic neurons. **c** Merged image of the BRP and TH channels. The scale bar is 50 μM. **d**-**f** show the average number of TH+ neurons counted in the DM, DL1 and DL2 dopaminergic clusters from larvae reared in control food (black bars) or nicotine food (red bars). The number of TH+ neurons was not affected by developmental nicotine exposure. Sample size was *n* = 19 brain hemispheres for control and *n* = 15 brain hemispheres for nicotine from *n* = 4 independent experiments. Student’s t-test was used to compare the control versus the nicotine condition

**Fig. 5:**
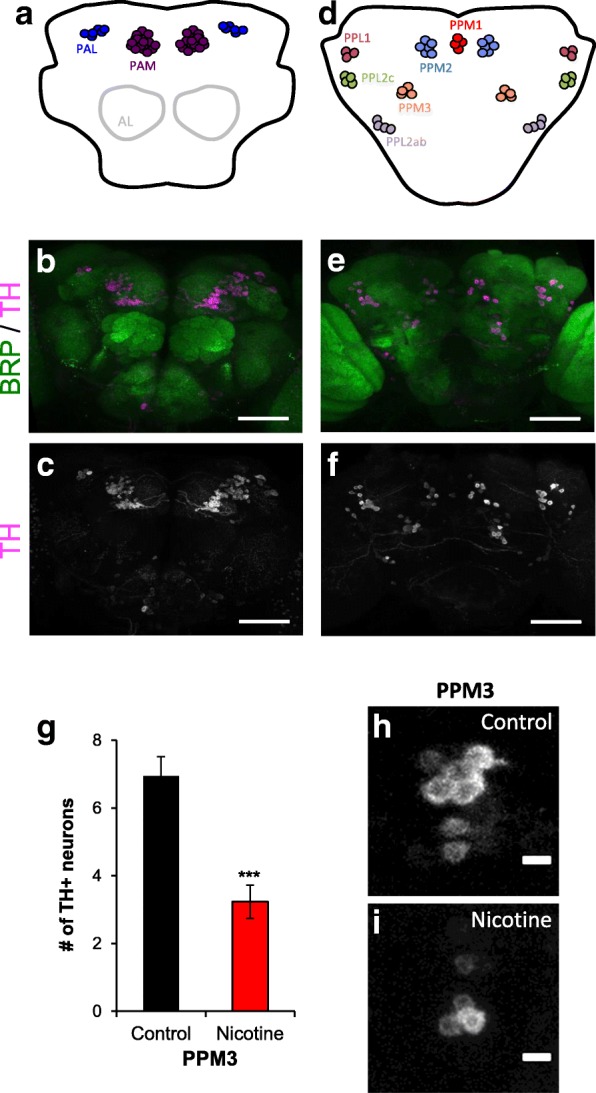
Developmental nicotine exposure decreases the number of TH+ neurons in the PPM3 cluster. Flies were reared on control or 0.3 mg/ml nicotine food. Adult brains were dissected, immunostained, mounted and imaged on a confocal microscope. **a**, **d** Schematic of the approximate position of the adult dopaminergic clusters. **a** Anterior clusters shown, **d** posterior clusters shown. **b**, **c** Maximum projection images of the anterior region of a brain from an adult fly that was reared in control food. The PAM and PAL dopaminergic clusters are visible. **e**, **f** are maximum projection images of the posterior region of the same brain. The PPM1, PPM2, PPM3, PPL1, PPL2ab and PPL2c are visible. **c**, **f** Anti-tyrosine hydroxylase (TH) was used as marker for dopaminergic neurons. **b**, **e** Anti-bruchpilot (BRP) was used as background staining for the whole brain. Merged images of the BRP and TH channels. Scale bar 50 μM. **g** Average number of TH+ neurons counted in the PPM3 cluster of flies reared in control food (black bars) or nicotine food (red bars). **h**, **i** Representative images of the PPM3 cluster from flies reared in control (**h**) or nicotine food (**i**). The number of TH+ neurons in the PPM3 cluster was reduced in brains from nicotine-exposed flies. Sample size was *n* = 20 brain hemispheres for control and *n* = 34 brain hemispheres for nicotine from *n* ≥ 5 independent experiments. Student’s t-test was used to compare the control versus the nicotine condition

**Fig. 6:**
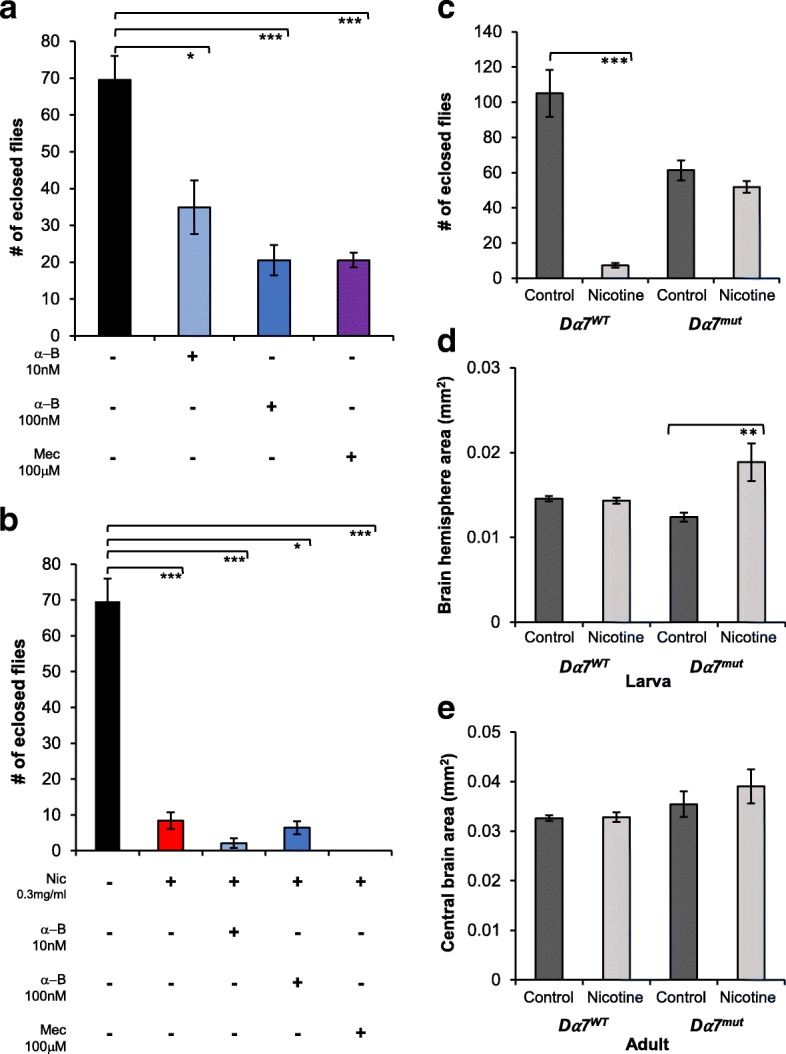
*Dα7* mediates the effect of nicotine on eclosion but not on larval brain size. **a**, **b** *w*B flies were reared on control (black bars) or 0.3 mg/ml nicotine (red bars) food and the number of eclosed flies was determined. **a** *w*B flies were reared in control food or food with nAChRs blockers: 10 nM (light blue bars) or 100 nM (dark blue bars) α-bungarotoxin (α-B) or 100μM (purple bars) mecamylamine (Mec); these drugs significantly decreased eclosion. **b** Blocking nAChRs during developmental nicotine did not revert the effect of nicotine. **c**-**e** *Dα7*^WT^ (dark grey bars) or *Dα7*^mut^ (light grey bars) flies were reared on control or 0.1 mg/ml nicotine food and the number of eclosed flies or brain size was determined. **c** *Dα7* mediates the effects of developmental nicotine exposure on eclosion. **d**, **e** *Dα7* does not regulate larval or adult brain size. **a**, **b** *w*B flies. Sample size: *n* = 14 vials, control food; *n* = 18, 0.3 mg/ml nicotine food; *n* = 12 vials each for 10 nM or 100 nM α-bungarotoxin, 100μM mecamylamine,100 nM α-bungarotoxin+ 0.3 mg/ml nicotine, 100μM mecamylamine + 0.3 mg/ml nicotine; *n* = 10 vials, 10 nM α-bungarotoxin+ 0.3 mg/ml nicotine; 2 independent experiments for nAChR blockers, 3 for control and nicotine food. **c** Sample size: *n* = 12 vials for *Dα7*^WT^ and *Dα7*^mut^, control food; *n* = 18, *Dα7*^WT^ nicotine food; *n* = 16, *Dα7*^mut^ nicotine food from 3 independent experiments. **d** Sample size: *n* = 10 larval brain hemispheres *Dα7*^WT^, control food; *n* = 10, 0.1/mg/ml nicotine food; *n* = 6 *Dα7*^mut^, control food; *n* = 11, 0.1 mg/ml nicotine food from 3 independent experiments, except *Dα7*^mut^ in control food, single experiment. **e** Sample size: *n* = 6 brains, *Dα7*^WT^ control food; *n* = 16, 0.1/mg/ml nicotine food; *n* = 7 *Dα7*^mut^, control food; *n* = 4, 0.1 mg/ml nicotine food from 2 independent experiments. **a**-**e** Kruskal-Wallis test, followed by pairwise comparisons adjusted for multiple comparisons

Nicotine was added to melted lab-prepared fly food to a final concentration of 0.3 mg/ml for experiments with the *w*B strain or 0.1 mg/ml for experiments with the EY5/EY6 strains (Sigma-Aldrich, N3876). Nicotinic acetylcholine receptors blockers, α-bungarotoxin (Tocris, 2133) or mecamylamine (Tocris, 2843) were added to either control food or nicotine food to the following final concentrations 10 nM α-bungarotoxin, 100 nM α-bungarotoxin, 100μM mecamylamine. Adult *wB* flies were crossed in vials (10 females and 4 males per vial for experiments in Figs. 1, 2, 3, 4 and 5; 7 females and 4 males per vial for experiments in Fig. 6) with the solidified control or nicotine food. The flies laid eggs for 2 days and then were removed from the vials. Their progeny was reared on either control or nicotine food from egg to 3rd instar larvae for larval brain experiments and from egg to 3–4 days after eclosion for adult brain experiments. Hence, “developmental” nicotine treatment for adult includes exposure during early life.

### Developmental assays

Flies began to eclose on Day 9 and continued to eclose up until Day 14, when we dissected them. During this time, the flies were kept in the vials they eclosed in, containing either nicotine or control food. To assess survival and eclosion delay, the number of newly eclosed flies were counted each day at the same time from day 9 to day 14, by marking empty pupae on the vial walls to indicate when a fly had eclosed. Survival was determined as the total number of flies that had eclosed by day 14. Eclosion delay was determined by calculating the ET50, the time it took for 50% of the total number of flies by day 14 in a given vial to eclose [50]. For experiments in Fig. 6, flies took longer to eclose when exposed to nicotine, so we extended eclosion data collection to day 16.

### Immunostaining

Larval brains and adult male fly brains were dissected in 1X Phosphate-Buffered Saline (PBS) pH 7.4 on day 7 and day 14, respectively. 3rd instar status was ensured by the distinctive branched spiracles of this stage before dissection, and not simply by day after egg laying. The sex of the larval animals used for dissections was not determined. Brains were fixed in 4% paraformaldehyde in 1X PBS for 30 min and then washed 3 times in 1X PBS for 10 min. The brains were incubated in blocking solution (5% goat serum in 1X PBS with 0.1% Triton-X100, PBT) for 2 h at room temperature on a rocker. Then the brains were incubated with primary antibodies, rabbit anti-tyrosine hydroxylase (Chemicon AB152, 1:400; [51, 52]) and mouse anti-bruchpilot (nc82, Developmental Studies Hybridoma Bank, 1:500; [53, 54]) diluted in blocking solution rocking overnight at 4 °C and washed 3 times in PBT for 30 min the next day. The following day, the brains were incubated with secondary antibodies on a rocker, goat anti-rabbit Alexa Fluor 594 (1:500; Invitrogen, Molecular Probes) and goat anti-mouse Alexa Fluor 488 (1:500; Invitrogen, Molecular Probes) diluted in blocking solution for 2 h at room temperature and washed 2 times in PBT for 20 min, and once more in PBS and mounted in Fluoromount-G (Southern Biotech, 0100–01). The immunostaining protocol used for Fig. 6 was slightly modified as follows: goat serum block incubation was 60 min, primary antibody incubation was at 4 °C for two nights without rocking. The secondary antibody incubation and mounting steps were the same, but were done 2 days after dissection.

### Confocal microscopy

Larval and adult brains were imaged with a laser scanning confocal microscope (Laser Scanning Microscope 710, Zeiss) using a 40X/1.3 DIC objective with oil immersion. These images were used to determine the number of neurons in individual dopaminergic clusters and also for central brain area and tyrosine hydroxylase (TH) fluorescence for adult brains. Additional images of larval brains were taken with the 10X/0.3 DIC objective to obtain images that included both brain hemispheres and a single background for both hemispheres. These images were used for larval brain hemisphere area and TH fluorescence measurements.

### Image analysis

Image analysis was done in Fiji ImageJ 1.51a. Damaged adult or larval brain hemispheres were excluded from analysis. Similarly, adult or larval brains in which we identified antibody penetration problems based on uneven staining between hemispheres and/or lack of staining in known clusters in control brains were excluded from TH positive (TH+) neuronal count and TH fluorescence level analysis. TH fluorescence levels were quantified within a region of interest drawn around the adult central brain or individual larval brain hemispheres. Total fluorescence of the TH staining within this region was normalized to background. TH levels are reported as “Corrected TH fluorescence,” which was determined by adapting a “corrected total cell fluorescence” procedure previously described [55, 56]. Briefly, integrated density, area, and mean grey value were measured on regions of interest with the “Measure” plugin of Fiji on maximum intensity projections with enhanced contrast by 0.4% so the edges of each brain were clearly visible. Background TH staining levels were determined on a rectangular region of interest outside the adult brain or larval brain. TH fluorescence level for larval brains was measured within a region of interest drawn around each hemisphere separately using the polygon selection tool. For adult brains, a region of interest was drawn around the central brain, excluding the optic lobes, using the freehand drawing tool. Brain fluorescence was measured in arbitrary units. We divided the corrected fluorescence by 1000, as noted on the Y-axis of the graphs depicting these results in Fig. 3. Dopaminergic clusters were identified according to Mao and Davis [52] for adult brains and Selcho et al. [57] for larval brains. Regions of interest were drawn on a maximum projection image of the brain to identify individual clusters. These regions of interest were saved and used as a frame within which neurons were counted for each cluster. Counts were done by eye, going through the confocal stack slowly to resolve when one neuron started and ended. Neurons were counted by using the multi-point tool with the smallest dot size and no labeling selected. Each image contrast was enhanced 0.001%, in order to see individual neurons. Images of brains from control or nicotine-exposed flies were counted blind.

### Statistical analyses

Values shown are mean ± Standard Error of the Mean (SEM). The figure legend text reports the number of samples, the number of independent experiments and the statistical test used for each data set. Sample size per experiment is reported as follows: *n* = number of samples per condition from *n* = number of independent experiments. “Sample” for each experiment is defined in the corresponding figure legend. Statistical comparisons were carried out in SPSS (version 24). Statistically significant differences at a significance level of *p* < 0.05 was used to determine differences between the control and the experimental condition(s). Data were analyzed with parametric or non-parametric tests depending on the result of the Levene’s test of equality of variance. If variances were not significantly different, Student’s t-test was used to determine differences between control and the experimental condition. If variances were significantly different, Mann-Whitney U-test was used. When more than two conditions were compared we used the Kruskal-Wallis non-parametric test followed by pairwise comparisons adjusted for multiple comparisons. Asterisks on graphs denote significance levels as follows; one for *p* < 0.05, two for *p* < 0.01, three for *p* < 0.001.

## Results

To determine the effects of nicotine on brain size and on the development of the dopaminergic system in the fruit fly brain, we exposed *Drosophila melanogaster* to nicotine during development from egg to larva to adult. We then used immunohistochemistry to visualize the brain and dopaminergic neurons in the brains of 3rd instar larvae and adult fruit flies, quantifying several features to determine the effects of developmental nicotine exposure (Fig. 1a).

To examine if developmental nicotine exposure has an effect on the dopaminergic system we first confirmed that the nicotine exposure protocol had been effective. It should be noted that developmental nicotine exposure was continuous from egg to 3rd instar larvae, or from egg to 3–4 days after eclosion. Hence, when we refer to “developmental nicotine exposure” for adult, this encompasses from embryo to early life. Developmental nicotine exposure has been previously shown to decrease survival and increase developmental time in *Drosophila melanogaster* [23]. Hence, we expected that flies with developmental nicotine exposure in our experiments would similarly have a significant decrease in the number of flies that survived to adulthood and exhibit developmental delay compared to flies grown on medium without nicotine. As anticipated, there was a significant effect of nicotine on survival and developmental delay (Fig. 1b, c). Fewer flies reached adulthood when reared on a medium containing nicotine (74.9 ± 3.1 eclosed flies in control vs 20.7 ± 1.8 in nicotine; *n* = 64 vials for control and *n* = 95 vials for nicotine; *p* < 0.001) and took significantly longer (11.5 ± 0.1 days to 50% eclosion in control vs 12.5 ± 0.1 in nicotine; *n* ≥ 64 vials; *p* < 0.001) to reach 50% eclosion (ET50). This indicates that the developmental nicotine exposure was effective.

### Developmental nicotine exposure increases larval brain area

Prenatal nicotine in mice has been shown previously to decrease newborn brain size, but this difference was no longer detected by postnatal day 10 [26]. Other studies have found an effect of prenatal nicotine exposure on brain size at postnatal day 21 [25]. We aimed to determine if developmental nicotine exposure can affect brain size in *Drosophila melanogaster* at two developmental stages, 3rd instar larvae and adult. Flies were reared in control food or nicotine food and collected at either 3rd instar larvae or 4 days after eclosion for adult flies. The brains were dissected and immunostained for bruchpilot (anti-BRP), which marks synaptic active zones throughout the brain [53, 54], and brain area was measured. Larval brain hemisphere area was significantly increased (0.021 ± 0.0007 mm^2^ in control vs 0.028 ± 0.0009 mm^2^ in nicotine; *n* = 35 brain hemispheres for control and *n* = 28 brain hemispheres for nicotine; *p* < 0.001) in flies reared on nicotine (Fig. 2a). However, we did not find statistically significant differences in central brain area (0.039 ± 0.0015 mm^2^ in control vs 0.036 ± 0.0012 mm^2^ in nicotine; *n* = 24 brains for control and 21 brains for nicotine; *p* = 0.194) at the adult stage (Fig. 2b). These results show that developmental nicotine exposure has an effect on brain size at the larval stage, but not at the adult stage in *Drosophila melanogaster*.

### Developmental nicotine exposure decreases overall tyrosine hydroxylase levels in adult brains but not larval brains

Prenatal nicotine has been shown to alter dopamine levels in the mammalian brain [28–32, 34]. Most of these studies in mammalian systems used a biochemical approach to detect changes in dopamine levels, measuring dopamine’s metabolites in whole brain or in brain region homogenates. In *Drosophila,* overall changes in neurotransmitter expression after drug exposure have been successfully detected using immunohistochemistry [50, 58]. Hence, we used an immunohistochemical approach to determine if developmental nicotine exposure has an effect on dopamine expression in 3rd instar larvae brains or adult fly brains from flies reared on control or nicotine food. We immunostained larval or adult brains for tyrosine hydroxylase (TH) in lieu of dopamine, because TH, the rate limiting enzyme for dopamine synthesis, is a marker for dopaminergic neurons in *Drosophila melanogaster* [52, 57]. Integrated density fluorescence levels were normalized to background, and reported as corrected TH fluorescence. Whereas TH levels at the larval stage were modestly elevated for larvae reared on nicotine (317.92 ± 22.94 fluorescence in arbitrary units in control vs 392.74 ± 36.61 in nicotine; *n* = 18 brain hemispheres for control and *n* = 22 brain hemispheres; *p* = 0.089) (Fig. 3a), TH levels were significantly lower in adult central brains from flies reared on nicotine food (278.24 ± 35.31 fluorescence in arbitrary units in control vs 190.75 ± 24.88 in nicotine; *n* = 10 brains for control and *n* = 17 brains for nicotine; *p* < 0.05) (Fig. 3b). These data show that developmental nicotine exposure at the concentration tested had no statistically significant effect in overall TH levels at the larval stage, but significantly decrease levels at the adult stage.

### Developmental nicotine exposure has no effect on the number of TH+ neurons in larval brains

The above results indicate that developmental nicotine exposure affects gross brain structure and overall TH expression at different developmental stages. Next, we examined changes in dopamine expression at the cellular level by determining the influence of developmental nicotine exposure on the number of neurons in individual dopaminergic nuclei. We counted all TH positive (TH+) neurons per cluster in larval brain hemispheres from flies reared on control or nicotine food. Larval brains were co-stained with an anti-TH antibody to visualize dopaminergic neurons and an anti-BRP antibody as background staining for the whole brain (Fig. 4a-c). Dopaminergic clusters DM, DL1, and DL2 were identified based on a previous characterization [57]. The number of neurons per cluster we counted in images of control brains was comparable to previously reported numbers for these clusters [57, 59]. Developmental nicotine exposure had no statistically significant effect on the number of TH+ neurons in the DM (11.4 ± 0.6 neurons in control vs 12.7 ± 1.4 in nicotine; *p* = 0.4), DL1 (7 ± 0.3 neurons in control vs 7 ± 0.6 in nicotine; *p* = 1) or DL2 (6.2 ± 0.4 neurons in control vs 5.9 ± 0.3 in nicotine; *p* = 0.6; for DM, DL1 and DL2 *n* = 19 brain hemispheres for control and *n* = 15 brain hemispheres for nicotine) clusters (Fig. 4d-f). These results show that developmental nicotine exposure did not affect the number of TH+ neurons at the larval stage.

### Developmental nicotine exposure decreases the number of TH+ neurons in the adult PPM3 cluster

The finding that nicotine exposure decreased overall TH fluorescence (Fig. 3b) suggests there may be changes in the number of TH+ neurons at the adult stage. Hence, we investigated if developmental nicotine exposure had an effect on the number of TH+ neurons in adult dopaminergic clusters. Adult brains were co-stained with an anti-TH antibody to visualize dopaminergic neurons and an anti-BRP antibody as background staining (Fig. 5). Dopaminergic clusters were identified based on a previous characterization [52]. The number of neurons we detected in brains from flies reared on control food were similar to what has been shown previously [52]. First, we quantified if developmental nicotine exposure changed the number of TH+ cells in the anterior dopaminergic clusters, PAM (Fig. 5a-c, 88.7 ± 3.3 neurons in control vs 84 ± 3.9 in nicotine; *p* = 0.5) and PAL (Fig. 5a-c, 4.9 ± 0.2 neurons in control vs 4.8 ± 0.2 in nicotine; *p* = 0.9). Our results show that there was no significant difference in the number of TH+ neurons in the anterior dopaminergic clusters between control and nicotine-treated brains. Next, we determined the effect of developmental nicotine exposure on the posterior dopaminergic clusters. We found that the number of TH+ neurons was not significantly different in nicotine-exposed flies in the PPM1 (Fig. 5d-f, 1.5 ± 0.2 neurons in control vs 1.7 ± 0.2 in nicotine; *p* = 0.4), PPM2 (Fig. 5d-f, 7.5 ≥ 0.6 neurons in control vs 6.6 ≥ 0.3; *p* = 0.2), PPL1 (Fig. 5d-f, 9.9 ± 0.3 neurons in control vs 8.3 ± 0.6 in nicotine; *p* = 0.2), PPL2ab (Fig. 5d-f, 5.3 ± 0.5 neurons in control vs 5 ± 0.3 in nicotine; *p* = 0.7), and PPL2c clusters (Fig. 5d-f, 1.6 ± 0.2 neurons in control vs 1.7 ± 0.1 in nicotine; *p* = 0.5). However, we detected a significant decrease in the number of TH+ neurons in the PPM3 cluster (Fig. 5d-i, 7 ± 0.6 neurons in control vs 3.2 ± 0.5 in nicotine; *p* < 0.001; *n* = 20 brain hemispheres for control and *n* = 34 brain hemispheres for nicotine for each of the adult dopaminergic clusters). These results show that developmental nicotine exposure had a cluster-specific effect in the adult dopaminergic system of the *Drosophila* brain.

### Role of nicotinic receptors on the effects of developmental nicotine exposure

To explore if some of the effects of developmental nicotine exposure described above were mediated by nicotinic acetylcholine receptors (nAChRs), we blocked these receptors in parallel with developmental nicotine exposure. We focused on the effect of nicotine on eclosion and larval brain area. If nicotine acted via nAChRs, we would expect to see a decrease in the effects of the nicotine treatment. We first took a pharmacological approach, using 100 μM mecamylamine, a non-specific blocker of nAChRs that has been used previously to block nAChRs in *Drosophila melanogaster* [60, 61]. However, the mecamylamine treatment was greatly detrimental to flies even without nicotine. *w*B flies had a 70% decrease in the number of eclosed flies, which was significantly different from control (Fig. 6a; 69.6 ± 6.4, *n* = 14 vials for control; 20.5 ± 2, *n* = 12 vials for mecamylamine, *p* < 0.001). As expected, *w*B flies exposed to 0.3 mg/ml nicotine food had decreased number of eclosed flies. Addition of 0.3 mg/ml nicotine to the mecamylamine food resulted in no survival (Fig. 6b; 8.4 ± 2.3 eclosed flies, *n* = 18 vials for nicotine, *p* = 0.001; 0.0 ± 0 eclosed flies, *n* = 12 vials for mecamylamine + nicotine; *p* < 0.001).

Given that mecamylamine had non-nicotine related effects on eclosion, we tested a more specific nAChR blocker, α-bungarotoxin, which binds to receptors that likely include the Dα5 and Dα7 subunits that have been shown to form heteromeric channels [62, 63]. Dα5, Dα7, and Dα6 have the highest similarity to vertebrate α7 subunits of nAChRs [64]. Moreover, an α-bungarotoxin sensitive nAChR has been shown to mediate the effects of nicotine on the startle response of adult flies [46]. We found that the two concentrations tested of α-bungarotoxin, 10 nM and 100 nM, were detrimental to *w*B flies when added to control food, causing a 50 and 70% decrease in the number of eclosed flies, respectively (Fig. 6a; 69.6 ± 6.4 eclosed flies, *n* = 14 vials for control; 34.9 ± 7.3 eclosed flies, *n* = 12 vials for 10 nM α-bungarotoxin, *p* = 0.021; 20.5 ± 4.1 eclosed flies, *n* = 12 vials for 100 nM α-bungarotoxin, *p* < 0.001). In addition, α-bungarotoxin (10 nM or 100 nM) with 0.3 mg/ml nicotine decreased eclosion to a similar extent as nicotine alone when compared to control (Fig. 6b, 69.6 ± 6.4 eclosed flies, *n* = 14 vials for control; 8.4 ± 2.3 eclosed flies, *n* = 18 vials for 0.3/mg/ml nicotine, *p* = 0.001; 2.1 eclosed flies ± 1.3, *n* = 10 vials for 10 mM α-bungarotoxin + nicotine, *p* < 0.001; 6.4 ± 1.7 eclosed flies, *n* = 12 vials for 100 mM α-bungarotoxin + nicotine, *p* = 0.016).

Since even the low concentration of α-bungarotoxin had deleterious effects on eclosion when added to control food, we switched to a genetic approach, testing whether effects of developmental nicotine would be reverted in a null *Dα7* mutant. We chose *Dα7*, because it was previously shown to mediate the effect of developmental nicotine exposure on survival and development time [23]. We used the previously characterized strains EY6, the *Dα7* mutant (*Dα7*^mut^) and its genetic control, EY5 (*Dα7*^WT^) [49]. These strains are more sensitive to nicotine than *w*B, so we performed experiments exposing these fly strains to 0.1 mg/ml nicotine in the food, as previously used [23]. As had been shown before, *Dα7*^WT^ flies were highly affected by the nicotine treatment (Fig. 6c, 105.1 ± 13.3 eclosed flies, *n* = 12 vials for *Dα7*^WT^ control food; 7.3 ± 1.3 eclosed flies, *n* = 18 vials for *Dα7*^WT^ nicotine food, *p* < 0.001), while the *Dα7*^mut^ flies were not significantly affected by nicotine at the 0.1 mg/ml concentration (Fig. 6c, 61.3 ± 5.6 eclosed flies, *n* = 12 vials for *Dα7*^mut^ control food; 51.9 ± 3.3 eclosed flies, *n* = 16 vials for *Dα7*^mut^ nicotine food, *p* = 1). These results support a role for *Dα7* in mediating the effects of developmental nicotine exposure on survival, measured as the number of eclosed flies and replicate previous findings [23].

Next, we asked if *Dα7* mediated the effect of developmental nicotine exposure on brain size. We reared *Dα7*^WT^ and *Dα7*^mut^ flies on control or 0.1 mg/ml nicotine food and dissected their brains at either the 3rd instar larval stage or the adult stage and measured larval brain hemisphere area or adult central brain area. We did not find statistically significant differences in larval brain hemisphere size between the *Dα7*^WT^ strain and the *Dα7*^mut^ strain when reared in control food (Fig. 6d, 0.014 ± 0.0003 mm^2^ area, *n* = 10 brain hemispheres for *Dα7*^WT^ control food; 0.012 ± 0.0005 mm^2^ area, *n* = 6 brain hemispheres for *Dα7*^mut^ control food, *p* = 0.138). The size of *Dα7*^WT^ larval brain hemispheres was not significantly affected by the nicotine treatment (Fig. 6d, 0.014 ± 0.0003 mm^2^ area, *n* = 10 brain hemispheres for *Dα7*^WT^ control food; 0.014 ± 0.0003 mm^2^ area, *n* = 16 brain hemispheres for *Dα7*^WT^ nicotine food, *p* = 1.0). Surprisingly, the brain hemispheres from *Dα7*^mut^ flies reared on nicotine food were larger than those from *Dα7*^mut^ flies reared on control food (Fig. 6d, 0.012 ± 0.0005 mm^2^ area, *n* = 6 brain hemispheres for *Dα7*^mut^ control food; 0.018 ± 0.002 mm^2^ area, *n* = 8 brain hemispheres for *Dα7*^mut^ nicotine food, *p* = 0.005). We did not detect any differences in central brain size either between *Dα7*^WT^ and *Dα7*^mut^ flies reared in control food, or with the nicotine treatment (Fig. 6e, 0.032 ± 0.0005 mm^2^ area, *n* = 6 brain hemispheres for *Dα7*^WT^ control food; 0.032 ± 0.002 mm^2^ area, *n* = 10 brain hemispheres for *Dα7*^WT^ nicotine food; 0.035 ± 0.0009 mm^2^ area, *n* = 7 brain hemispheres for *Dα7*^mut^ control food; 0.039 ± 0.003 mm^2^ area, *n* = 4 brain hemispheres for *Dα7*^mut^ nicotine food).

These results confirm previous findings that *Dα7* mediates the effect of developmental nicotine exposure on survival. Our findings suggest that *Dα7* is not involved in regulating normal brain size at neither the 3rd instar larva nor the adult stages. These results also suggest that activation of nAChRs that do not contain *Dα7* subunits by developmental nicotine play a role in regulating larval brain size.

## Discussion

Our results demonstrate that *Drosophila melanogaster* can be a model organism for the study of a variety of effects of developmental nicotine exposure. While prior work showed a nicotine-induced decrease in survival, delayed development and decreased sensitivity to acute nicotine and ethanol exposure by adulthood [23], here we demonstrate gross structural changes in the nervous system and cellular effects following developmental exposure to nicotine. Specifically, we found that developmental nicotine exposure caused an increase in brain hemisphere area of larval brains without affecting brain size in adults. Nicotine also caused a decrease in TH staining fluorescence at the adult stage, but no changes at the larval stage. In addition, nicotine caused a decrease in the number of TH+ neurons in one adult dopaminergic cluster, PPM3, without significantly changing any other adult nuclei or any of the larval dopaminergic clusters. Lastly, we validate a role for *Dα7* in promoting decreased survival during nicotine exposure and identified a possible role for non*-Dα7* containing nAChRs in mediating the effect of developmental nicotine on larval brain size.

### Effect of prenatal nicotine exposure on brain size

Here we show that developmental nicotine exposure increased brain area in larval brains by 30%, but this difference did not persist to adulthood in *Drosophila melanogaster* (Fig. 2). There is precedent for an increase in brain size as the outcome of prenatal nicotine exposure in Long Evans rats at postnatal day 21 [25]. However, this effect differed in females versus males. While there was an increase in brain size for females, a decrease was observed in males [25]. This was the only stage tested, so it is not known whether this effect was present earlier in development and persisted, nor whether this effect would have disappeared at later stages. The present study did not distinguish between female and male larvae. It would be interesting to determine whether developmental nicotine exposure has different effects in females versus males in a follow up investigation.

Other studies have also reported decreases in brain size in mice [26]. However, there are variations in the stage or stages of development when the decrease in brain size was observed. For example, one study showed that prenatal nicotine exposure in mice decreased brain weight and brain length at birth. However, these differences did not persist and were no longer detected at postnatal days 10, 20, or 50 [26]. A different study in Sprague-Dawley rats showed reductions in weight for specific brain regions (midbrain/brainstem, cortex, and cerebellum) after prenatal nicotine exposure that could be detected over several postnatal time points, but were no longer detected by the 5th week of postnatal development [65]. Our data coincides with these studies in that we show that brain size is affected by developmental nicotine exposure at early stages of development, but this difference is no longer detected later in development. The differences in the specific developmental stage at which developmental nicotine exposure had an effect on brain size, the duration of this effect, and whether the effect observed was an increase or decrease in brain size may be due to variations in nicotine exposure protocol, nicotine dose, strain or species differences, or stage of development tested. Despite these disparities in specific outcome, it can be concluded that developmental nicotine exposure affects brain size early in development in multiple species.

The molecular mechanisms underlying the changes in brain size are not well understood. Slotkin et al., (1987) proposed that the decrease in brain region weight they showed is part of an overall delay in development caused by prenatal nicotine exposure, which is resolved given enough time for the pups to catch up [65]. However, the molecular mechanisms for this developmental delay are not well known. Studies focusing on changes at the cellular level have identified changes in cellular composition after prenatal nicotine exposure, with an increase in glia and a decrease in neuronal area coupled to an increase in neuronal density [24, 66]. These results point towards a mechanism involving regulation of cell differentiation and proliferation.

### Effect of prenatal nicotine exposure on dopamine levels

Our results show that TH levels were decreased by developmental nicotine exposure in *Drosophila melanogaster* in adult flies. No difference was found at the 3rd instar larval stage (Fig. 3). These results are consistent with studies that have found a correlation between prenatal nicotine exposure and decreases in dopamine levels. However, previous studies have shown disparities in the effects of prenatal nicotine exposure on dopamine levels depending on the region of the brain studied and the developmental stages studied. One study found a reduction in dopamine content in rat cerebral cortex after prenatal nicotine exposure that was largest after birth and dissipated by weaning [28]. This study did not include fetal stages. Another study measured dopamine levels after prenatal nicotine exposure and reported no change in dopamine levels in rat forebrain at gestational day 18, an increase in dopamine levels by postnatal day 15, and no difference by 10 weeks of age [29]. A different study found no change in dopamine levels in frontal cortex after prenatal nicotine exposure at postnatal day 22 [30]. However, this study showed changes in dopamine levels in other brain regions including the striatum, nucleus accumbens, VTA, and substantia nigra that either decreased or increased depending on the nicotine concentration tested and whether or not the pups had developed hyperactivity after prenatal nicotine exposure [30]. Contrasting these results, another study found no differences in dopamine levels in the striatum nor hypothalamus, but decreased dopamine levels in neocortex, and midbrain-postmedulla in brains from young-adult rats euthanized at 8–9 weeks of age [31]. A more recent study in mice showed an increase in baseline dopamine levels in frontal cortex, but no difference in the striatum at postnatal day 42 after prenatal nicotine exposure [32]. Also in mice, a couple studies by a different group reported decreased dopamine levels in prefrontal cortex of mice at postnatal days 28 and 56 by HPLC and extended these results by measuring baseline dopamine levels by microdialysis in mice at postnatal days 56 to 63 [33, 34]. As the studies referenced above show, the exact effects of developmental nicotine exposure reported in the literature vary, possibly because of differences in the mode of nicotine exposure, strains and species differences, the developmental stage or stages tested, and the brain region analyzed. However, our results coincide with these studies in the fact that developmental nicotine exposure has been shown to affect dopamine levels in the brain.

The mechanisms underlying the effect of developmental nicotine exposure in dopamine levels are not well understood. Alkam et al. [34] showed a decrease in TH+ varicosities in prefrontal cortex, which they associate with decreased dopamine levels measured by microdialysis. These results suggest that the decrease in dopamine levels could be due to the changes in dopamine expression at the cellular level, either changing the number of TH+ varicosities per neuron or changing the number of TH+ dopaminergic neurons. However, these quantifications were not reported [34].

Taken together these studies suggest the effect of prenatal nicotine exposure on dopamine levels reflects changes at the cellular level that either affect the number of cells that express dopamine or the number of neuronal varicosities they have. Future research could focus on identifying how nicotine regulates dopaminergic neuron proliferation, specification and differentiation.

### Effect of prenatal nicotine exposure on the number of TH+ neurons

The present study quantified the number of TH+ neurons in brains from flies reared on control food or nicotine food and found a decrease in the number of TH+ neurons in one adult cluster, PPM3, 4 days after eclosion, which is early adulthood for *Drosophila melanogaster.*

Few mammalian studies have quantified changes in the number of dopaminergic neurons after prenatal nicotine exposure. One study quantified the number of TH+ cells in the substantia nigra and VTA of adult rat brains after prenatal nicotine exposure and found no effect at postnatal days 75 or 82 [67]. Alkam et al. [34], referenced in the previous section, did not count the number of TH+ neurons, but focused instead on the number of TH+ axonal varicosities of midbrain dopaminergic neurons after prenatal nicotine exposure in mice brains at postnatal day 44. They counted fewer TH+ varicosities in prefrontal cortex and the nucleus accumbens, but no changes in the striatum after prenatal nicotine exposure. The decrease in axonal varicosities may have reflected a decrease in TH+ neurons, but this was not investigated.

Other studies have shown changes in the number of dopaminergic neurons in rats after prenatal or perinatal exposure to other compounds, such as glucocorticoids [68, 69]. These studies suggest that the number of dopaminergic neurons is highly plastic. Additionally, the notion of dopaminergic neurons as a plastic population has been shown before in the context of research on activity-dependent neurotransmitter specification. Several studies have reported changes in the numbers of dopaminergic neurons that are activity-dependent [70–72].

In *Drosophila melanogaster*, we found no effect of prenatal nicotine exposure at the larval stage. However, one adult dopaminergic cluster, PPM3, was affected by prenatal nicotine exposure. PPM3 is a protocerebral posterior medial dopaminergic cluster with main neuropil projections going to a structure in the *Drosophila* brain called the central complex [52]. The central complex is a neuropil involved in locomotion, memory for visual objects, sleep, startle-induced arousal, wakefulness, and aggression [73–79]. Of these behaviors, dopamine, and specifically neurons of the PPM3 cluster have been involved in ethanol-induced locomotion, wakefulness, and aggression [73, 74, 79]. The PPM3 cluster has been shown to have fewer TH+ neurons in *Drosophila* models of Parkinson’s disease [80, 81]. However, there have been discrepancies in reports showing changes in dopaminergic neurons in *Drosophila* Parkinson’s disease models [82]. Navarro et al. [82] did not find statistically significant changes in the number of dopaminergic cells, but found significant decreases in dopamine levels, assessed via a GFP reporter under the control of the TH-promoter. Kong et al. [73] also show evidence that the PPM3 cluster has a role in a drug-induced behavior, ethanol-induced locomotion, making this cluster a good candidate for mediating behavioral effects of developmental nicotine exposure. Taken together, these studies suggest that dopaminergic neurons, including the PPM3 cluster, in *Drosophila* are a plastic population.

We did not determine whether the decrease in PPM3 neurons observed with developmental nicotine exposure resulted from loss of TH or loss of the neurons in the PPM3 cluster region. There could be two different mechanisms at play 1) neurotoxicity, resulting in cell death, which would resemble a Parkinson model, or 2) neurotransmitter plasticity, in which the neurons would switch neurotransmitter expression to a different one. There is evidence that dopaminergic neurons can up or downregulate TH expression within specific dopaminergic clusters after chronic activation [72]. It has also been shown that dopaminergic neurons co-express other neurotransmitters, such as NPY and GABA [70, 71]. Activity-dependent neurotransmitter switching is a well- documented phenomenon [83, 84]. It will be interesting to determine which mechanism underlies the decrease in PPM3 TH+ neurons after developmental nicotine exposure.

### Involvement of nAChRs on the developmental nicotine effects on eclosion and brain size

Acetylcholine is the main fast neurotransmitter in insects and *Drosophila* has 10 genes coding for nAChR subunits [64, 85–87]. Nicotine acts on nAChRs, which are linked to cell proliferation, differentiation, maturation and survival in nervous system development [88]. We blocked nAChRs to determine their possible role in the effects of developmental nicotine exposure, but blocking nAChRs with the unselective blocker mecamylamine or with the more selective blocker α-bungarotoxin resulted in significant decreases in survival (Fig. 6a). Moreover, blocking nAChRs during developmental nicotine exposure affected eclosion at the same level as the nicotine treatment alone (Fig. 6b). However, we noticed a 70% decrease in eclosion between 100 nM α-bungarotoxin on its own and 100 nM α-bungarotoxin with nicotine, compared to 94% decrease in eclosion between 10 nM α-bungarotoxin on its own and 10 nM α-bungarotoxin with nicotine. This suggests that the higher α-bungarotoxin dose slightly ameliorated the effect of developmental nicotine exposure on eclosion, which could indicate that nAChRs blocked by α-bungarotoxin mediate the effects of nicotine on eclosion.

α-bungarotoxin has been shown to bind to the Dα5 subunit, which can form heteromeric channels with Dα7 subunits [62, 63]. In *Drosophila*, *Dα7* was previously shown to be involved in the effect on survival and developmental delay caused by developmental nicotine exposure [23]. Our results match those findings, as there was no difference in eclosion between *Dα7*^mut^ flies reared in control or nicotine food (Fig. 6c). Hence, *Dα7* is needed for the decreased survival upon developmental nicotine exposure displayed by flies with wildtype *Dα7.*

Next, we tested if *Dα7* mediated the effect of developmental nicotine on brain size. Our results suggest that *Dα7* is not involved in regulating normal brain size, given than flies without a functional copy of the gene, *Dα7*^mut^, had larval or adult brains with similar size to the brains from the genetic control strain *Dα7*^W*T*^ (Fig. 6d, e). We did not see an effect of developmental nicotine at the 0.1 mg/ml concentration in the *Dα7*^WT^ strain. The lack of effect of developmental nicotine on *Dα7*^WT^ larval brains may be explained by the differences in nicotine dose used in each set of experiments or on genetic background between *w*B flies and the *Dα7* strains. For example, larval brain hemisphere size of wB and *Dα7*^WT^ was considerably different (0.021 mm^2^ vs 0.014 mm^2^, respectively). It should be noted that *Dα7*^WT^ flies are very sensitive to nicotine exposure, with a 93% decrease in eclosion on nicotine food. Thus, treatment survivors may be immune to some of the effects of developmental nicotine treatment through de novo mutations. However, *Dα7*^mut^ had about a 50% increase in larval brain hemisphere area after developmental nicotine exposure, suggesting that *Dα7* is not necessary for the nicotine-induced increase in larval brain size. It follows that nicotine activation of other nAChRs that do not contain Dα7 might be sufficient to convey the signal to increase larval brain size. It would be interesting to determine the specific contributions of different nAChRs in future investigations.

## Conclusions

Nicotine abuse is a major health problem worldwide and despite widespread knowledge of the consequences of smoking many women continue smoking during pregnancy or are exposed to nicotine via nicotine replacement therapy or e-cigarette use. Current research is addressing the developmental changes induced by nicotine at the genetic, molecular and cellular levels, and how those changes result in long-term behavioral consequences.

We have shown that developmental nicotine exposure in *Drosophila melanogaster* affects the dopaminergic system. These effects were detected at a nicotine concentration that in flies has been shown to affect normal development and to decrease their sensitivity to acute exposure to nicotine and ethanol by adulthood. Overall, we found changes in brain area, TH levels and number of TH+ neurons that were stage-specific and cluster-specific. Developmental nicotine exposure increased 3rd instar larval brain hemisphere area, decreased overall TH levels in adult brains, and decreased the number of TH+ neurons in the PPM3 adult dopaminergic cluster. We also confirmed previous results that *Dα7* mediated the effects of developmental nicotine on survival, while it does not seem to play a role in normal brain size. Our results suggest non-Dα7 receptors mediate the effect of nicotine in larval brain size.

These changes in the dopaminergic system could have consequences in behaviors mediated by this neurotransmitter system, such as reward and drug responses. Additionally, alterations to specific dopaminergic nuclei have been reported in the mammalian brain after prenatal nicotine exposure, albeit the underlying mechanisms for these alterations are not known. There is conservation at the genetic and molecular level in developmental processes from *Drosophila* to higher order organisms. Developmental studies in mammals take a longer time and are more costly than developmental studies in fruit flies. A *Drosophila* model for developmental nicotine exposure will be most valuable for contributing to elucidate genes and signaling pathways that lead to the effects of nicotine that have been described. *Drosophila* is a powerful model organism to study the effects of drugs. Given the specific effects of developmental nicotine exposure we have characterized on the dopaminergic system further research in the underlying genetic, cellular, and molecular mechanisms for developmental nicotine exposure in *Drosophila* will offer a tractable option for faster and complimentary discovery to what has been achieved using other model systems.

## Abbreviations

Dα5: Drosophila nicotinic acetylcholine receptor alpha 5 subunit
Dα6: Drosophila nicotinic acetylcholine receptor alpha 6 subunit
Dα7: Drosophila nicotinic acetylcholine receptor alpha 7 subunit
Dα7^mut^: Drosophila alpha 7 mutant strain, EY6
Dα7^WT^: Drosophila alpha 7 genetic control strain, EY5
BRP: Bruchpilot
DIC: Differential interference contrast
DL1: Dorso Lateral 1 larval dopaminergic cluster
DL2: Dorso Lateral 2 larval dopaminergic cluster
DM: Dorso Medial larval dopaminergic cluster
ET50: Time to 50% eclosion
nAChR: Nicotinic acetylcholine receptor
PAL: Protocerebral Anterior Lateral
PAM: Protocerebral Anterior Medial
PBS: Phosphate-Buffered Saline
PBT: PBS with 0.1% Triton-X100
PPL1: Protocerebral Posterior Lateral 1 dopaminergic cluster
PPL2ab: Protocerebral Posterior Lateral 2ab dopaminergic clusters
PPL2c: Protocerebral Posterior Lateral 2c dopaminergic cluster
PPM1: Protocerebral Posterior Medial 1 dopaminergic cluster
PPM2: Protocerebral Posterior Medial 2 dopaminergic cluster
PPM3: Protocerebral Posterior Medial 3 dopaminergic cluster
SEM: Standard Error of the Mean
TH: Tyrosine hydroxylase
TH+: TH positive
*wB*: *w*^*1118*^ Berlin
